# Sport-Related Concussion: Evaluation, Treatment, and Future Directions

**DOI:** 10.3390/medsci7030044

**Published:** 2019-03-15

**Authors:** Lydia McKeithan, Natalie Hibshman, Aaron M. Yengo-Kahn, Gary S. Solomon, Scott L. Zuckerman

**Affiliations:** 1 Vanderbilt Sports Concussion Center, Vanderbilt University School of Medicine, Nashville, TN 37232, USA; lydia.j.mckeithan@vanderbilt.edu (L.M.); natalie.hibshman.1@vanderbilt.edu (N.H.); a.yengo@vumc.org (A.M.Y.-K.); gary.s.solomon@vumc.org (G.S.S.); 2 Department of Neurological Surgery, Vanderbilt University Medical Center, Nashville, TN 37232, USA

**Keywords:** sport-related concussion, mild traumatic brain injury, genetics, neuroimaging, biomechanics

## Abstract

Sport-related concussion (SRC) is a highly prevalent injury predominantly affecting millions of youth through high school athletes every year. In recent years, SRC has received a significant amount of attention due to potential for long-term neurologic sequelae. However, the acute symptoms and possibility of prolonged recovery account for the vast majority of morbidity from SRC. Modifying factors have been identified and may allow for improved prediction of a protracted course. Potential novel modifying factors may include genetic determinants of recovery, as well as radiographic biomarkers, which represent burgeoning subfields in SRC research. Helmet design and understanding the biomechanical stressors on the brain that lead to concussion also represent active areas of research. This narrative review provides a general synopsis of SRC, including relevant definitions, current treatment paradigms, and modifying factors for recovery, in addition to novel areas of research and future directions for SRC research.

## 1. Introduction

The centers for disease control (CDC) estimate that 1.6 to 3.8 million sports related traumatic brain injuries (TBI) occur every year [1]. This is widely regarded as an underestimation, as many people suffering from mild TBI (mTBI) do not seek medical attention [2]. Several studies have also demonstrated increasing incidence of sport-related concussion (SRC) in recent decades [3,4]. 

Not surprisingly, SRC and mTBI have become topics of major public health interest. While graver concerns have been raised regarding the proposed long-term adverse effects of SRC and repetitive head trauma, current evidence remains insufficient to support a causal association between sport-related head trauma, long-term neurocognitive dysfunction, and neuropathological sequela [5,6,7,8]. Despite these broadcasted concerns, the vast majority of morbidity related to SRC occurs acutely during the first weeks to months following injury, thus a basic foundational understanding of SRC management and modifying factors is paramount for all healthcare providers treating this injury.

The purposes of the current narrative review focused on sports concussion are to: (1) explain the definition of SRC and associated terminology; (2) summarize recommended evaluation and treatment methods; (3) assess modifying factors that contribute to the incidence and severity of SRC; and (4) discuss new areas of SRC research, including genetics, advanced neuroimaging, and helmet engineering.

## 2. Definitions

### 2.1. Sport-Related Concussion

According to the Concussion in Sport Group’s (CISG) 5th international conference, held in Berlin in October 2016, the current definition of an SRC involves the following four criteria [9]:

- A direct or indirect trauma anywhere on the body with a force transmitted to the head;
- Rapid (seconds to minutes) or delayed (minute to hours) symptom presentation, typically with spontaneous resolution;
- Negative standard neuroimaging (computerized tomography (CT) or magnetic resonance imaging (MRI)), reflecting a functional rather than structural injury;
- With or without loss of consciousness, with stepwise resolution of symptoms.

It is important to define the overlap between SRC and mild traumatic brain injury (mTBI). The CDC defines a TBI as, “a bump, blow, or jolt to the head, or a penetrating head injury that disrupts the normal function of the brain.” Severity is intertwined with the Glasgow coma scale (GCS) score, ranging from “mild” (GCS 13–15, a brief change in mental status or consciousness) to “moderate” (GCS 9–12), to “severe” (GCS 3–8, an extended period of unconsciousness or amnesia after injury). Simply stated, concussion overlaps with mTBI, with a GCS score of 13–15. Where the two differ is that according to the CISG, any positive imaging can no longer be classified as a concussion (irrespective of a GCS score of 13–15) and is referred to as mTBI if the clinical exam equates to a GCS of 13–15. Colloquially, concussion is considered a form of mTBI, and the terminology is often used interchangeably.

### 2.2. Post-Concussion Syndrome

While the majority of SRCs are resolved within 2–3 weeks, symptoms may persist for months. This clinical scenario is most aptly termed post-concussion syndrome (PCS). However, the definition of PCS varies considerably. Post-concussion syndrome was included in many earlier versions of the *Diagnostic and Statistical Manual of Mental Disorders* (DSM), only to be removed from the current version [10]. The lack of agreed upon definition poses a challenge when interpreting research studies. Rose et al. surveyed 597 sports medicine physicians and found that the most common time course to diagnose PCS clinically was 1–3 months (33%), with a minimum of at least one symptom (56%). Though the time course of 1–3 months was most common, a true majority opinion did not exist, indicating the heterogeneity of PCS. [11] For consistency and the purposes of standardization, we support and utilize the generally accepted definition of PCS as at least one symptom lasting more than 30 days after SRC [11].

## 3. Evaluation and Treatment

### 3.1. Immediate Management

Once an athlete is suspected of having an SRC, they are removed from play and precluded from returning to play for at least the remainder of the day. Slogans such as, “*When in doubt, sit them out*,” have become ubiquitous, reinforcing the zero-tolerance nature of no same day return to play [12]. As indicated by the above slogan, the initial diagnosis is made by a medical provider, usually sideline-based certified athletic trainers (ATCs), and is largely driven by clinical signs and symptom endorsement. Diagnosis is helped further by enlisting someone familiar with the player’s baseline personality and affect in order to judge deviations from baseline. Evaluation protocols remain variable, and several tests are commonly used in tandem with clinical judgment. These sideline tests include: modified balance error scoring system (mBESS); vestibular/ocular motor screening (VOMS), post-concussion symptom scale (PCSS), and elements of the lengthier sports concussion assessment tool 5 (SCAT5), which includes both the mBESS and PCSS. Various clinical domains are evaluated with these sideline tests (Table 1). Most importantly, for an initial diagnosis, no single, gold-standard test exists, and even widely-accepted tests have questionable reliability, such as the mBESS, with which groups have been found to have high variability and large number of errors [13]. Multiple areas of SRC sign and symptom endorsement should be evaluated, which requires an individualized approach to each athlete and injury.

**Figure 1:**
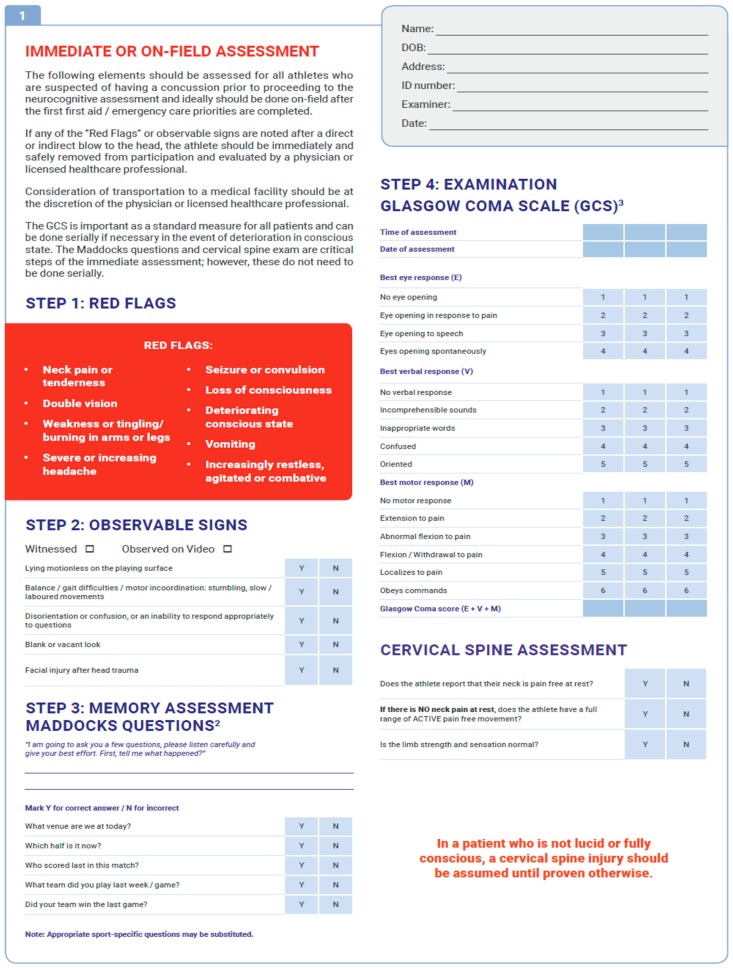
One page of the SCAT5 (please find the figure at the bottom of the manuscript). Reproduced from sport concussion assessment tool—5th edition with permission from BMJ Publishing Group Ltd. doi.org/10.1136/bjsports-2017-097506SCAT5.

A standardized framework helps guide providers in balancing the subjective elements of SRC diagnosis. Efforts to adopt a uniform protocol began in 1997 with the standard assessment of concussion (SAC) [14], which measures orientation, immediate memory, concentration, and delayed recall, and, when compared with a baseline score, has been found to have high sensitivity and specificity for diagnosing SRC [15,16]. Today, perhaps the most well-known and comprehensive evaluation is the sports cognitive assessment tool (SCAT), which has undergone several iterations to optimize its power and utility. Of note, the SCAT includes the aforementioned SAC. The SCAT5 is the most recent version and starts with an immediate on-field assessment, which sequentially involves identification of red flags warranting immediate medical attention, observable signs, orientation assessment (Maddocks questions), GCS, cognitive assessment (SAC), postural stability, and a cervical spine assessment (Figure 1). The entire SCAT5 is lengthy and not administered entirely on the sideline, but elements can be used immediately, saving the rest for a more controlled environmental setting (e.g., locker room or office examination room). 

Sport-related concussion diagnosis is based on clinical judgment in tandem with the data gathered from the previously mentioned assessment tools. A player can have multiple signs and symptoms to as few as one and still be diagnosed with an SRC. In these scenarios, no substitute exists for clinical experience, training, and a sound knowledge of each athlete’s baseline personality and behavior. 

### 3.2. Off-the-Field Assessment

Once an initial diagnosis is made, the athlete is restricted from significant exertional activity until SRC signs and symptoms resolve. Resolution of signs and symptoms then lead to protocols involving returning to school, followed by return to sport. Once the diagnosis has been made, most concussions are monitored by an ATC or other medical professional, including primary care providers, sports medicine physicians, neurologists, neuropsychologists, neurosurgeons, or nurse practitioners. Ideally, the medical provider has access to computerized neuropsychological testing to provide another objective measure of the player’s change from baseline [17]. To best utilize this methodology, players should complete a symptom checklist along with neuropsychological and postural stability tests prior to the start of a season to establish an objective baseline, which can then be compared to post-injury performance [18]. The immediate post-concussion assessment and cognitive testing (ImPACT) test is one of the most widely used neurocognitive assessment tools and has been demonstrated to have high sensitivity and specificity for detecting concussion and monitoring recovery [19]. However, even this robust neurocognitive battery is not used in isolation, but rather with a blend of other clinical evaluation tools during assessment. 

### 3.3. Recovery

Return to play (RTP) is the resumption of athletic participation following return to neurologic baseline. It is suggested that this occur after return to learn (RTL), which is resumption of classroom learning with tolerable or resolved symptoms. The RTP process suggested by McCrory et al. and the CISG is a stepwise process, outlined below in stages [9].

- Stage 1: Activity limited by symptoms: introduction of daily activities that do not provoke symptoms.
- Stage 2: Light aerobic exercise of low intensity: elevation of heart rate above baseline activity with actions such as walking or cycling at a leisurely pace.
- Stage 3: Exercise specific to sport: begin sport-specific movement such as running; contact strictly avoided.
- Stage 4: Training without contact: resume drills with continued strict avoidance on contact with the goal of resuming coordination.
- Stage 5: Resumed full contact practice: participate in practice drills including contact. Close monitoring is suggested.
- Stage 6: Full return to play: resume normal participation in the sport.

For youth athletes, each stage lasts for 24 h before progression onto the next stage. In the United States, progression through the RTP steps is often mandated by state law. Should symptoms be provoked at any stage, there is ceasing of activity and a day of rest, then a subsequent resumption of the previous stage [9]. Return to learn is accomplished in the first stage, suggesting that the cognitive baseline should be reached before beginning to incorporate exercise into the recovery [20].

### 3.4. Treatment

The general consensus on treatment is that athletes should rest for 1–2 days, both mentally and physically, following an SRC to minimize energy demands to the brain and allow post-concussive symptoms to resolve. Athletes should take a more active approach to recovery, gradually increasing their physical and cognitive activity level as much as possible without experiencing recurrent symptoms. A 2015 consensus meeting was held in Pittsburgh to address the complex topic of recovery after SRC [21], and several important conclusions were drawn. First, limited empirical evidence exists for prescribed physical and cognitive rest after SRC, and such strategies may not be effective. While this is a recognized gap in empirical literature, physicians continue to use their best judgement on the matter. Second, strict brain rest, formerly referred to as “cocoon” therapy, is not recommended and may have negative effects on recovery. Third, early evidence suggests that active rehabilitation may improve symptom recovery more than prescribed rest alone, and this calls for matching specific treatments to individual clinical profiles. Examples of active rehabilitation include immersing athletes in enriched environments (intellectual activities and social networks), submaximal aerobic therapy, treadmill exercises, visualization training, and light coordination activity [22]. The amount of rest needed typically varies and there is insufficient evidence to propose an exact amount needed for recovery [9]. 

Unfortunately, there are no targeted pharmacologic treatments for concussion symptoms. Pharmacotherapy can be directed at symptom management, but the CISG suggests that RTP should be assessed in the absence of medical intervention, so as not to mask the SRC symptoms and ability of the patient to proceed through the steps.[9] A full discussion of targeted pharmacologic therapy is outside the scope of this review, yet several informative summaries exist by Solomon and Sills [23] and Petraglia [24] in adults, and Halstead [25] in the pediatric population.

## 4. Modifying Factors

Below we review common factors that may modify outcomes after SRC. When healthcare professionals are aware of these important factors that may be associated with a protracted recovery, they are better equipped to counsel patients and families and focus efforts on shortening recovery time or preventing unnecessary complications.

### 4.1. Initial Symptoms

Numerous studies have shown that one of the strongest predictors of a longer recovery is symptom severity after the initial injury [26,27,28]. Miller et al. evaluated a group of 294 patients presenting to a concussion clinic and found that athletes with a SCAT2 symptom severity score >20 were 8.7 times more likely to suffer from PCS (odds ratio (OR) 8.67, 95% confidence interval (CI) 2.43–30.8) compared to other significant factors associated with PCS, which all had lesser effect sizes [29]. In addition to symptoms, signs related to postural instability [30,31] and immediate memory deficits have been similarly predictive of a longer recovery [32]. Other studies have shown that on-field amnesia [33] and oculomotor dysfunction [34] are associated with greater total symptom scores. In a prospective cohort study of 531 patients presenting to a sports concussion clinic, Meehan et al. found overall symptom burden to be the most significant predictor of prolonged symptoms after SRC, with 28 day symptom resolution time for 86% of patients with post-concussion symptom scale (PCSS) < 13 compared to only 35% of patients with PCSS ≥ 13 [35]. Given the compelling evidence from these key studies [24,25,26,27,28,29,30,31,32,33], we can reasonably conclude that more symptoms and more severe symptoms at time of injury suggest a longer recovery.

### 4.2. Concussion History

A prior concussion appears to increase the risk of experiencing a future concussion. A study evaluating 2905 collegiate football players found that players reporting ≥3 SRC are three times as likely to have another SRC (OR 3.0, 95% CI 1.6–5.6) compared to those without a prior history [36]. In terms of recovery time, the evidence is mixed regarding whether a prior concussion is associated with prolonged recovery after a subsequent concussion [37,38,39]. Inconsistencies in study design and the characterization of recovery may account for this discrepancy. Terwilliger et al. demonstrated that in 42 adolescent athletes, those who sustained an additional significant impact within 24 h of the initial SRC had significantly longer length of recovery [40], suggesting that the time between subsequent concussions may influence recovery profiles and should be taken into account in future studies. 

### 4.3. Sex

Sex has been found to play a role in the incidence, symptom profile, and recovery of SRC. Although TBI rates in general are higher for males than females [41], numerous studies show that female athletes suffer higher rates of SRCs than their male counterparts in same-sex sports (relative risk RR, 1.7, 95% CI, 1.4–2.0) [42,43,44]. Female athletes are also more likely to have symptoms persist for a longer duration [45] and report a greater number of post-concussive symptoms [44]. Baseline symptom and neurocognitive profiles have also been shown to vary between genders, as female athletes have been shown to report more cognitive, emotional, and sleep symptom clusters and have higher verbal memory scores compared to men [46]. Conversely, a number of studies have found the relationship between sex and symptom profile or recovery not to be statistically significant [47,48]. Chrisman et al.’s retrospective cohort study evaluating 1412 high school athletes did not find sex to be a significant risk factor for concussive symptoms lasting ≥ one week for non-football sports (RR 1.1, 95% CI 0.7–1.6, *p =* 0.81) [49]. Variability in findings demonstrate the complexity of how males and females experience concussion differently. Several clinical and laboratory studies also demonstrate that estrogen and progesterone levels may play a neuroprotective role following mTBI [50], suggesting that females have a protective factor against concussion, which adds further complexity to the argument. This warrants further study to evaluate for more definitive relationships between sex and outcome.

### 4.4. Migraine Headaches

A personal or family history of migraine headaches has been associated with susceptibility to PCS. Zemek and colleagues reported that of 2584 pediatric patients presenting to the emergency department for concussion (and had adequate follow-up), personal migraine history was associated with recovery time of 28 days or longer (OR 1.9, 95% CI 1.4–2.6, *p* < 0.001) in the subset of 801 patients with a protracted course [28]. Of note, in the Zemek et al. study, no correlation between family history of migraine and concussion recovery was seen. Morgan et al., however, found in a retrospective study of 40 patients with PCS, compared with control patients without persistent symptoms, that family history of migraine and PCS development were positively correlated (*p =* 0.003), while personal history had no statistically significant association (*p =* 0.088) [37]. These findings suggest that some underlying susceptibility to migraine headaches may predispose athletes to a longer recovery, and there may be utility in treating migraines while recovering from an SRC. Perhaps the most authoritative conclusion on the topic of migraines comes from Iverson et al., where a systematic review of on predictors of recovery after SRC was conducted [51]. First, the authors concluded that a history of treatment for headaches or migraines was associated with more symptoms at baseline than those without. Second, having a preinjury history of migraines might be associated with a risk of longer recovery; however, only one study showed this association [28], albeit a large, multisite, and prospective mentioned earlier. The authors also noted that the study of migraines is complicated by difficulty differentiating between a headache history vs. migraine history. The direct interaction between SRC, PCS, and migraine remains exploratory until further investigation.

### 4.5. Learning Disability or Attention Deficit Hyperactivity Disorder

The literature evaluating the effect of learning disability (LD) on SRC susceptibility and recovery is inconsistent in showing strong and replicable correlations. Drawing from the exhaustive review by Iverson et al., [48] the authors concluded that those with attention deficit hyperactivity disorder (ADHD) or with LD had a greater lifetime risk of concussion, performed more poorly on baseline neuropsychologic testing, and reported more concussion symptoms at baseline. However, aside from incidence and baseline measures, the literature on the topic of recovery time and symptom duration was mixed. In their pediatric population of SRC and non-SRC subjects, Zemek et al. found that a history of LD was associated with symptom prolongation (OR 1.5, 95% CI 1.0–2.1 *p =* 0.03) [28]. In reference to ADHD, one pediatric study found that in 294 concussed athletes, ADHD was associated with symptoms lasting more than 28 days compared to athletes without ADHD (OR 3.87, 95% CI 1.13–13.24) [29]. Another investigation of 70 patients with a self-reported diagnosis of ADHD compared to 70 control patients without showed that ADHD athletes needed 16.5 days to recover compared to 13.5 to those without, though this was not statistically significance (*p =* 0.12) [52]. Despite these individual studies showing longer symptom duration, Iverson and colleagues concluded (based on an analysis of prospective studies only) that those with ADHD or LD were not at greater risk for worse or longer recovery after SRC [51].

### 4.6. Psychiatric Comorbidities

It is thought that psychiatric comorbidities may have an impact on recovery from SRC. One study of 569 concussed pediatric patients, 171 of which were due to sports, assessed the importance depression, anxiety, bipolar disorder, suicidal attempts, or post-traumatic stress disorder [53]. Guerriero et al. [53] found that having one such psychiatric condition before concussion significantly prolonged recovery time, with an average of 235 days in the premorbid positive psychiatric group and 143 days in controls (*p =* 0.03). A similar retrospective study of middle and high school athletes found that a premorbid psychiatric diagnosis was found to have a threefold increased chance of developing PCS (OR 3.1, *p =* 0.026) [37]. Returning to the systematic review by Iverson et al. [51], the authors similarly concluded that those with pre-existing psychiatric diagnoses, specifically depression, were at increased risk for longer symptom duration, and were also found to be more symptomatic at baseline. 

It appears that personal history is not the only factor that is associated with longer recovery time. Morgan et al. also showed that a family history of psychiatric condition increased the patient’s risk of longer recovery times and PCS [37]. A retrospective investigation by Legarreta et al. of 151 high school concussed athletes compared risk of PCS development in those with family or personal psychiatric histories to those without. Athletes with a family history alone were five times more likely (OR 5.06, 95% CI 1.71–14.99, *p =* 0.018) to develop PCS, while athletes with both family and personal history were about 2.5 times more likely (OR 2.52, 95% CI 1.20–5.30, *p =* 0.03) to develop PCS. Additional complexity exists for management of patients harboring comorbid psychiatric diagnoses, indicating a possible genetic factor in this subgroup of student-athletes. 

## 5. Future Directions

The study of SRC continues to evolve, and three examples of areas of active research include genetic sequencing, advanced neuroimaging, and equipment engineering. These exciting avenues have widened our understanding of individualized risk factors, pathophysiological mechanisms of injury and recovery, and biomechanical contributions to concussion.

### 5.1. Genetics

The body of genotype and SRC data is promising. Researchers are beginning to pinpoint genetic modifiers and explore how these factors influence susceptibility to injury and timing of recovery. With associations between SRC and genetics, future research may prove to have predictive value for SRC outcomes and influence medical management. Though several studies report positive associations, it is equally important to report the nonexistent associations between candidate genes and recovery in an effort to counteract the effect of publication bias and only reporting statistically significant results. 

#### 5.1.1. Interleukin 6 Receptor

Once such genetic association includes the cytokine interleukin 6 (IL-6), which is linked to an array of functions, such as regeneration and metabolism, but is most well-known as a hallmark for inflammation. Though not directly applicable to SRC, research on IL-6 in general TBI populations has laid the groundwork for mTBI and SRC studies. One study concluded that an increased level of IL-6 after severe TBI corresponded with worse outcome, with nearly 25% of living patients at six months suffered from severe cognitive disability [54]. Conversely, Chiaretti et al. collected cerebrospinal fluid from 29 children with TBI and found that increased IL-6 and nerve growth factor corresponded to better neurological outcome [55]. Notably, the differences between severe TBI and SRC are numerous, and while promising, future exploration into IL-6 and recovery from SRC specifically is warranted. 

Signaling through different IL-6 receptors (IL-6Rs) elicit pro and anti-inflammatory responses via trans and classical pathways, respectively [56]. In certain cases, there is a propensity for one common single nucleotide polymorphism (SNP), IL-R Asp358Ala, to coincide with a skewed proportion of soluble versus membrane bound IL-6R [57], increasing the pro-inflammatory response [58]. One study by Terrell et al. sought to explore the effect of the Asp385Ala SNP on outcome after SRC. After evaluating 1056 collegiate athletes, 133 of which suffered a SRC, the authors concluded that the Asp385Ala SNP increased risk of SRC compared to the population without this SNP [59]. These findings begin to demonstrate the complex biomolecular underpinnings of concussion and recovery and warrant further exploration.

#### 5.1.2. Apolipoprotein E e4

Another notable genetic association in concussion exists with the apolipoprotein E (APOE) class of proteins. This class of proteins is responsible for lipid transport, as well as structural moderation in mitochondria and dendrites [60]. Notably, APOE proteins are implicated in the transport of cholesterol to manage the nerve myelination in the brain [61]. Within the class of APOE proteins, three types of APOE proteins exist, namely APOE e2, APOE e3, and APOE e4. Of these, APOE e4 has been linked across various studies with poor outcome following concussion. One study reported that of 57 college athletes with SRC, 40% of those with the APOE e4 allele had more significant post-concussion impairment in a neurocognitive assessment compared to only 16.5% athletes without the APOE e4 allele (*p =* 0.046). Standardized pre-concussion scores showed no significant difference between APOE isotypes (*p =* 0.149) [62]. 

Though the literature leans favorably on APOE e4 as a negative prognostic factor in SRC, there have been studies that suggested otherwise. In a longitudinal study by Moran et al. exploring APOE e4 in children with mTBI, the ability of APOE e4 to predict poor or favorable outcomes was inconsistent [63]. This could support a theory that the lipid transport and neuromodulation after concussion in the pediatric population is different from that of adults. Another study found that having the APOE e4 allele decreased the risk of suffering a concussion in college athletes by up to 40% [59]. With the incongruities of reported data on APOE e4 concussion risk, outcome, and symptom severity, future studies should aim to investigate each of these variables in the same or more similar populations [64]. 

In a recent study by McCrea et al., the authors conducted a systematic review on genetic predictors of SRC [64]. A total of six studies compared preseason to postinjury gene expression and found several differences in immune, inflammatory, and hypothalamic-adrenal-pituitary pathways. More relevant to our discussion, four papers evaluating candidate genes were reviewed. These studies showed longer recovery times—in addition to symptoms and headache severity in some studies—for athletes with a variant of the variable number of tandem repeat (VNTR) allele, the APOE e4 allele, and the rs74174284 polymorphism in the promoter region of the SLC17A7 gene [64]. However, the authors mentioned that the risk of bias was high due to small sample size, poor representation of age, gender, sport, poorly defined diagnostic methods, referral bias, and lack of control group in the form of non-contact athletes. The overall level of evidence behind genetic associations in SRC was low. Though the role of genetics is exciting and may uncover important information, as of now, these study limitations and conflicting results preclude their use as a clinical tool in management and treatment. 

### 5.2. Advanced Medical Imaging

Due to the absence of abnormal findings on standard clinical imaging, attention has been shifted to advanced imaging techniques that may detect functional injuries. As shown by Giza and Hovda [65], the exact biochemical mechanism of concussion is thought to be due to an initial influx of ions and glutamate release leading to major energy demands, putting the brain in a period of metabolic crisis. Potassium efflux and sodium and calcium influx occur due to lipid membrane damage, which can trigger other voltage- or ligand-gated ion channels, leading to a diffuse spreading, depression-like state that clinically causes post-concussion impairment, which includes migraine symptoms, vulnerability to repeat injury, and cognitive impairment [65]. To restore ionic and cellular hemostasis, significant energy is needed for membrane ionic pumps to go into overdrive, which all occurs in the setting of reduced cerebral blood flow. This leads to a mismatch between energy supply and demand [65]. Additionally, calcium is driven intracellularly and is further sequestered into mitochondria. Lastly, intracellular oxidation and reduction states are altered, which generates damaging free radicals, making us susceptible to repeat injury. This impaired metabolism lasts up to 7–10 days. While an in-depth discussion of the neurometabolic cascade of concussion [66,67] is outside the scope of this narrative review, these mechanisms represent what is seen in advanced imaging studies and warrant a cursory review. 

With this metabolic cascade in mind, diffusion tensor imaging (DTI) and magnetic resonance spectroscopy (MRS) are two techniques often used to identify microstructural changes, such as axonal injury or neurotransmitter disruption, respectively. These changes reflect overall neuronal integrity and have the potential to be used as biomarkers in the evaluation and management of SRC [9].

#### 5.2.1. Diffusion Tensor Imaging

Diffusion tensor imaging (DTI) measures the direction and restriction of diffusion of water molecules, which is reflective of several white matter (WM) microstructural characteristics, such as myelination, axon diameter, fiber density, and organization. Fractional anisotropy (FA) and mean diffusivity (MD) are the two parameters typically obtained from DTI. The FA value, reported as a number between 0–1, defines the ratio of the direction of maximal diffusion to the diffusion that is perpendicular to that main direction [68]. Fractional anisotropy can decrease because of increased perpendicular diffusivity of water, thought to be caused by demyelination, or decreased parallel diffusivity, thought to be caused by axonal destruction [69,70,71]. 

Most studies report decreased FA and increased MD in subjects with mTBI [72,73]. Yet, there is conflicting evidence in studies evaluating change over time [74], with several reports showing elevated FA levels in the acute phase following concussion [75,76], and others showing elevated FA levels in the chronic phase as well [77]. Fractional anisotropy has also been found to correlate with a spectrum of acute symptoms and symptom duration [78]. In a cohort of adolescents with SRC, DTI imaging within two months showed that whole-brain FA levels correlated with the total SCAT2 score [79]. Thus, FA may hold promise as a potential biomarker of concussion severity and recovery as advances in imaging techniques, technology, and interpretation continue. 

Although many DTI studies focus on FA, there is evidence that MD may be a more sensitive marker for detecting mild changes in mTBI, suggesting FA is better at detecting structural injury in more severe cases of TBI [80,81]. Cubon et al.’s study of 10 college athletes with SRC revealed significantly increased MD in several left hemispheric clusters for all concussion subjects, but only significantly decreased FA for subjects with moderate to severe injury when compared to controls. Another study evaluating DTI parameters in 23 patients with mTBI found that MD was the only diffusion variable significantly changed for mTBI patients compared to controls. Their report also showed increased MD in long association WM tracts in concussed individuals with symptoms at three months compared to those who had recovered clinically.[81] Further study is necessary to identify a predictable pattern of FA or MD changes that occur over time and changes that can be related to specific clinical symptoms. 

As recent reviews have highlighted, major limitations of all DTI and advanced neuroimaging studies include small sample sizes, absences of baseline measures, and multiple statistical comparisons without adequate control for experiment-wise error rate [64]. The clinical significance of the positive associations found is also unknown. In a prospective study of high school and collegiate athletes participating in contact sports by Klein and colleagues [82], 138 concussed athletes were scanned up to four times and compared to non-concussed athletes, 135 of whom participated in contact sports and 96 in non-contact sports. Concussed athletes were more likely to have positive MRI changes relative to contact (30.4% vs. 15.6%, OR = 2.32, *p =* 0.01) and non-contact control athletes (19.8%, OR = 2.11, *p =* 0.04), and one concussed athlete had an acute, structural injury-related finding. The authors concluded that <1% of SRCs are associated with acute injury findings on qualitative structural MRI, which led to the recommendation that MRI was not indicated routinely following SRC. The results of this large, prospective study add context to the smaller studies finding significant associations. 

#### 5.2.2. Magnetic Resonance Spectroscopy

Magnetic resonance spectroscopy identifies and quantifies brain metabolites based on the frequency at which they resonate relative to a reference standard. Metabolites commonly measured with long echo times include *N*-acetyl aspartate (NAA), a marker for neuronal integrity [83], creatine (Cr) for cellular energy/attenuation, choline (Cho) for membrane turnover [84], and lactate for anaerobic metabolism. Metabolites measured with short echo times include glutamate/glutamine (Glx), which are excitatory amino acids released after brain injury [85], and myo-inositol, thought to be a marker of astroglial proliferation.

The data on MRS in SRC is limited, but drawing from the larger, general mTBI population, the most commonly reported findings in mTBI include lower NAA and higher Cho levels [86,87]. Many studies report decreased NAA in the acute phase of mTBI and a recovery of NAA over the course of 30 days. Interestingly, follow-up studies have shown a sustained decrease in NAA up to six months post-injury in those with a second concussion. This protracted NAA recovery time could be attributed to the additive stress of subsequent head impacts. Early MRS studies established correlations between decreased NAA and poor neuropsychological function following TBI in general [88,89], and newer evidence supports this association in concussion as well. Henry et al.’s study of 12 concussed athletes demonstrated a correlation between NAA and Glu changes in primary motor cortices and self-reported symptom severity (NAA, r = −0.554, *p =* 0.008 and Glu, r = −0.637, *p =* 0.001) [90]. This further demonstrates that neuronal integrity, as measured by NAA on MRS, has the potential to be quantified through advanced imaging and used as a marker of concussion severity and recovery. 

Fewer studies have looked at Glu or Gln levels in the brain as a marker of neurotransmitter metabolism and shown decreased levels following mTBI [83,90,91]. Creatine is often used as an internal standard and metabolite levels are reported as a ratio relative to Cr, such as NAA or Cr. Gasparovic et al.’s study also found significantly higher Cr levels in mTBI subjects when compared to healthy controls [83]. Thus, Cr might not be as stable as once thought in mTBI, and thus, could be confounding conclusions made about NAA changes in concussion. Furthermore, many of these studies were plagued by small sample sizes, did not obtain baseline (pre-injury) values, enlisted only pediatric populations, and focused on multiple brain regions, making it difficult to draw conclusions about neurometabolic changes in the general SRC population. 

#### 5.2.3. Functional Imaging Techniques

Functional MRI (fMRI) uses blood-oxygen-level-dependent (BOLD) contrast which detects increased local cerebral blood flow in areas undergoing increased neuronal activity. Altered BOLD signal in concussed individuals while performing a variety of neurocognitive (such as working memory and attention) and sensorimotor tasks has been shown [92]. Studies have also demonstrated increased neuronal recruitment in the dorsolateral prefrontal cortex (DLPFC) of concussed individuals, suggesting possible brain reorganization or neural compensation in response to the neuronal injury caused by mTBI [93]. Chen et. al. showed reduced activation in DLPFC in concussed individuals with poorer performance on neurocognitive tasks and depression [94,95], suggesting that a lack of neuronal recruitment following concussion may be associated with poor neurocognitive and neuropsychiatric outcomes. Several resting state fMRI studies of concussed athletes showed reduced functional connectivity in several neural networks in the acute and chronic periods following mTBI [93,96]. Murdaugh et al. interestingly found resting state fMRI hyper connectivity in posterior brain regions (left cerebellum and precuneus, and right middle occipital gyrus, *p =* 0.047, <0.0001, 0.001, respectively) and hypoconnectivity in anterior brain regions (right inferior frontal gyrus (IFG), middle frontal gyrus (MFG), middle temporal gyrus (MTG), and posterior cingulate, *p =* 0.005, 0.05, 0.013, 0.01, respectively) in high school football players with SRC compared to those without [97]. More recent reviews of resting state fMRI in SRC have shown that changes in functional connectivity have the potential to predict SRC recovery profiles and clinical outcomes. Madhavan et al. revealed that functional connectivity was significantly correlated with symptom severity score (r = −0.28, *p =* 0.002, n = 91) and that decreased connectivity < 10 days post injury was associated with symptom severity score at a later point (3 weeks to 3 months *p* < 0.05) [98]. 

#### 5.2.4. Clinical Significance vs. Imaging

Advanced neuroimaging has great potential to detect the acute microstructural and functional changes proposed to occur with SRC. However, with the inconsistency of current evidence and the variability of advanced imaging protocols across institutions, there is still much more to ascertain before we can have confidence in the clinical utility of these imaging techniques. Numerous reports have also used advanced neuroimaging techniques to study potential long-term outcomes of SRC [99,100,101,102]. Many of those studies report significant p-values when comparing athletes who have experienced concussion to controls, but have failed to demonstrate convincing effect sizes or clinically meaningful differences. A recent systematic review revealed the possibility of type 1 error in most of those studies, which often make a large number of statistical comparisons and find only a small fraction of them to be statistically significant [103]. Two additional systematic reviews explored acute and long-term neuroimaging findings in SRC and found underwhelming results, citing the use of multiple comparisons and lack of correlation with clinical measures [103,104]. The acute study [104] found that 8 of 11 studies conducted a total of 809 comparisons of brain function, of which 149 (18%) were statistically significant, and the chronic study [103] found that 13 of 16 studies made a total 456 comparisons of brain activity, of which 171 were statistically significant (38%). Thus, caution should be taken when extrapolating conclusions from studies that take the liberty of making multiple comparisons or do not report objective clinical measures. 

### 5.3. Helmet and Engineering

Many engineering groups are studying ways to prevent or minimize the occurrence of SRC, particularly in helmet design. Helmets used in contact sports were originally designed to prevent severe life-threatening outcomes, such as skull fracture or hemorrhage. Since then, our understanding of helmet technology has grown immensely [105,106]. 

Sport-related concussion is thought to be due to a combination of linear acceleration, which causes transient increases in intracranial pressure, and rotational acceleration, which causes a microstructural strain response in brain tissue [107]. While helmets are able to dissipate linear acceleration, they are less able to reduce rotational acceleration, which is thought to be the primary entity implicated in concussion [107,108]. Although several companies market helmets that claim to protect against concussion, a lack of published scientific literature exists, due in part to the proprietary nature of the products of helmet companies. Studies have been published that evaluate several innovations, such as tethering a helmet to shoulder pads [109] or introducing an additional layer on top of a helmet, but show limited scientific evidence supporting the clinical benefit of implementing these new designs. Specifically, Breedlove and colleagues evaluated the Guardian Cap and demonstrated that the device reduced force at velocities high enough to cause skull fracture, but failed to mitigate impact forces at the low and medium velocities typically involved in concussion [110]. One study designed a helmet prototype that significantly reduced rotational acceleration with the addition of an outer shell [111], suggesting a potential promising direction for future helmet design. Further study is necessary to evaluate how implementation of various designs may affect the incidence of concussion during real-time play. Rowson and Duma at Virginia Tech conducted a great deal of research on laboratory testing of helmets and developed the STAR rating system [112], which can be a helpful tool for assessing performance of future helmet designs.

The use of helmet accelerometers is also germane to helmet technology. Since the study of forces experienced by a player’s brain in real-time is limited, controlled laboratory simulations are used as substitutes to evaluate internal forces experienced during a head impact [113]. Helmet embedded accelerometers have been used in efforts to identify a force threshold for concussive impacts [114] but have been proven largely unreliable [9]. Accelerometers are often used at different locations, and significant heterogeneity in accelerations exists [115]. Wu and colleagues compared common sensor locations and concluded that mouthguard sensors had the best skull coupling when compared to mastoid patches and skull caps [116]. Nonetheless, accelerometers and high-speed video technology are only able to provide external measurements that are difficult to accurately translate to localized internal effects, such as focal strain and deformation [117,118]. However, further research has been aimed at developing computer-based models that can predict the forces experienced by brain tissue foci during various impacts and have potential utility in the study of future helmet design [119].

## 6. Conclusions

While concussion has been extensively studied, increased public awareness of SRC in recent decades has driven scientific research of the mechanisms underlying mTBI and discovery of diagnostic and prognostic tools. Significant progress has been made in the evaluation and management of SRC, with much yet to be learned in determining the exact physiologic changes that occur with mTBI and characterizing recovery among the numerous individuals suffering from SRC. With the technology at hand to take a more critical look at concussion research, opportunity exists to not only extract these mechanisms, but to disentangle their connections with elements like modifying factors described here. The advancement is ongoing, and we are hopeful that the genetics and engineering lenses can shed light on the future management of SRC.

## Figures and Tables

**Table 1:** Recommended assessment and suggested tests in the acute evaluation of sport-related concussion (SRC).

Domain	Suggested Test(s)
Postural Stability	Modified balance error scoring system (mBESS)
Oculomotor Functions	Vestibular/ocular motor screening (VOMS)
Neurocognitive Functions	Standard assessment of concussion (SAC) of the sport concussion assessment tool-5th edition (SCAT5)
Symptoms	Post-concussion symptom scale (PCSS) of the sport concussion assessment tool-5th edition (SCAT5)
